# TB Data Improvement in Nkembo Health Treatment Center in Libreville, Gabon

**DOI:** 10.3390/tropicalmed11040090

**Published:** 2026-03-27

**Authors:** Casimir Manzengo, Farai Mavhunga, Nlandu Roger Ngatu, Fleur Lignenguet, Stredice Manguinga, Ghislaine Asseko Nkone

**Affiliations:** 1 World Health Organization (WHO), Ouagadougou BP 7019, Burkina Faso; 2 World Health Organization (WHO), 1202 Geneva, Switzerland; mavhungaf@who.int; 3 Department of Public Health, Kagawa University Faculty of Medicine, Kagawa 761-0701, Japan; ngatu.nlandu@kagawa-u.ac.jp; 4 Programme National Tuberculose (PNT), Libreville BP 50, Gabon; fleurlegendes@gmail.com (F.L.); manguingas@gmail.com (S.M.); 5 World Health Organization (WHO), Libreville BP 820, Gabon; nkoneassekog@who.int

**Keywords:** tuberculosis, data review, Gabon, National TB Program

## Abstract

Although the estimated tuberculosis (TB) incidence in Gabon is declining, there have been challenges with treatment coverage, HIV status and treatment outcome documentation. Thus, the National TB Program (NTP) conducted an innovative data review at the Nkembo Health Treatment Center in Libreville, which manages more than 70% of Gabonese TB patients. Since our hypothesis was that the Nkembo treatment center was struggling with data mismanagement due to the workload, the objective was to perform a TB data quality review and triangulation exercise at the Nkembo health facility in Libreville, from January to August 2023, and propose recommendations for data improvement. Methods: The study used the data reconciliation method. This is a process that involves comparing and aligning data from multiple sources to ensure consistency, accuracy, and integrity. The primary purpose of data reconciliation is to identify and resolve discrepancies or differences between datasets and make them consistent. Using the “TB onion model”, analysis identified data mismanagement as a key contributor to underreporting. A data review compared TB records to TB registry data and patient folders from January to August 2023 for notification and to the 2022 cohort for treatment results. The study focused on notified TB cases, HIV status and TB treatment outcome documentation. Discrepancies were reconciled, and treatment outcomes re-evaluated. Results: After review, statistically significant increases were observed: +22% for total TB cases (*p* = 0.0003), +141% for the number of TB cases with known HIV status (*p* = 0.0017) and +104% for the number of TB cases successfully treated (*p* = 0.0001), as compared with the previous data. Discussion: This data reconciliation showed the usefulness of triangulation across data sources to improve the completeness of data. Also, current reported data underestimate the number of reported cases, documentation of HIV status, and treatment success. Conclusions: The study shows that data reconciliation can improve TB programmatic data completeness to better reflect program performance.

## 1. Introduction

Tuberculosis (TB) is a preventable communicable infectious disease caused by *M. tuberculosis.* It is a major global health issue in the Sub-Saharan Africa region (SSA) and was reported to be the leading cause of death from a single infectious agent prior to the 2019 coronavirus disease (COVID-19) outbreak [1]. With more than 10 million people continuing to fall ill with TB every year and more than 1 million dying from the disease, the recent WHO report that covered more than 99% of the world’s population ranked tuberculosis (TB) the world’s leading cause of death from a single infectious agent and among the top 10 causes of death worldwide in the year 2025 [2]. Globally, TB incidence slightly decreased from 10.8 million to 10.7 million in 2024, as compared to the previous year. Similarly, TB mortality decreased from 1.27 million to 1.23 million in the same period. Although some regions of the world have achieved substantial reduction in TB burden, the disease incidence has increased in low- and middle-income countries (LMICs). Undernutrition, diabetes, alcohol consumption, smoking and HIV infection are reported to be major risk factors for TB. In addition to the above-mentioned factors, extreme poverty and homelessness are also associated with TB incidence and mortality in Gabon [2,3].

In Gabon, TB remains a major public health problem, with the capital city of Libreville bearing the heaviest burden. Despite international support by the Global Fund, the Gabonese National Tuberculosis Program (NTP) faces various challenges, including low TB coverage (42%) and low success rates (57%). In 2022, the HIV seropositivity rate among TB patients was 29%, and only one-third of them underwent HIV testing, according to the NTP annual report [4]. The TB response in Gabon is coordinated by the Minister of Health through the National Program, known as NTP, which was established in 1997. Since 2004, the program has been jointly funded by the Gabonese Government and the Global Fund. Despite this support, the program’s performance has been suboptimal on key program indicators. While the incidence has been slowly declining from 542/100,000 in 2014 to 513/100,000 in 2022, the estimated number of deaths caused by TB has increased from 1974 to 4039 related deaths in the same period [5].

There have also been concerns regarding the programmatic performance trends over recent years. For example, the treatment coverage declined from 49% in 2019 to 42% in 2022, whereas the proportion successfully treated declined from 67% in 2019 to 57% in 2022 (2021 cohort). Furthermore, the proportion of TB patients with a documented HIV status declined from 38% in 2019 to 24% in 2022. At the end of 2022, almost 60% of expected TB cases were not notified, half of the confirmed patients were not successfully treated, and the HIV status of more than three-quarters was not documented [5]. According to the WHO Global tuberculosis report, the most important risk factors in Gabon are HIV and malnutrition [2]. In fact, the HIV prevalence is 3.6% in the general population, and it was estimated at 28% in TB patients. Only 6.4% of PLWHA on antiretroviral therapy are benefiting from TB preventive treatment [6]. Furthermore, given the Purchasing Power Parity (PPP) data, Gabonese people are facing the high cost of a healthy diet (approximately $4.64 per day), and 37% of the population cannot afford it. The high prevalence of undernutrition (25%) exacerbates vulnerability to communicable infectious diseases such as TB, which negatively affects the disease prognosis [7].

To better understand and improve the situation, the WHO supported the Gabonese NTP in performing periodic data reviews to assess data quality and adopt some data quality improvement strategies. The national annual TB report stated that 78% of TB patients in Gabon were diagnosed in Libreville in 2022, and within this town, a large majority of cases were treated at the Nkembo health facility. These data helped to focus on this health facility, which has the highest number of TB patients, for data review.

High-burden facilities may face TB data management issues in relation to data completeness, accuracy, and consistency. Obviously, facility-level data are useful for evidence-based planning, including budgeting, staffing, and supply chain management, as well as regular analysis of core indicators, which contribute to strengthening TB surveillance. Thus, the “WHO standards and benchmarks for TB surveillance and vital registration” guide countries in evaluating and improving TB surveillance systems [8]. It is a tool that helps to assess national TB surveillance systems’ ability to accurately measure TB cases and deaths, identify gaps that should be addressed to improve the surveillance system, and define the actions needed to strengthen TB surveillance in countries where gaps were identified [8]. This suggests the importance of training healthcare workers in data analysis and interpretation for sustaining the improvement of TB data surveillance.

Objectives

1. Perform a quality review of TB surveillance data related to patients treated at the Nkembo health facility in Libreville from January through August 2023;
2. Identify the key issues related to the data quality with direct impact on the reporting process;
3. Formulate the recommendations to improve data recording and reporting in this health facility, as well as patient management.

## 2. Methodology

The Nkembo health facility is one of the healthcare settings in Libreville specialized in TB management. The services offered include TB laboratory diagnosis, case management, psychosocial support and HIV testing. It is the biggest of the 12 healthcare settings that provide TB management services in Libreville. Given that approximately 70% of all TB cases in Gabon are treated at the Nkembo health facility, suboptimal performance at this facility has significant implications for the overall performance at the national level.

### 2.1. TB Onion Model Theory of Change

**Figure 1:**
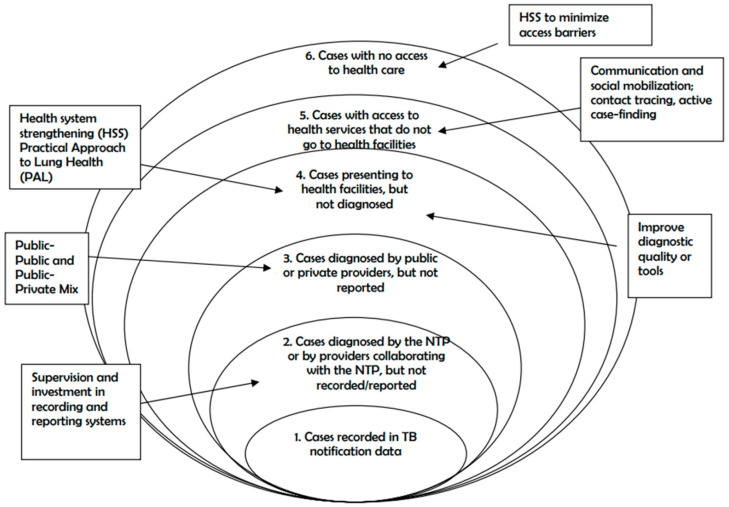
The “onion” model: A framework for assessing the fraction of TB cases accounted for in TB notification data, and how this fraction can be increased.

In this review, we used the TB onion change model [9] as a conceptual framework to help to understand why many TB cases are not reported at the national level as shown in the Figure 1.

### 2.2. Study Team

The review team was composed of 15 people and implemented in four days. The reviewers carried out this task after their regular work to avoid disturbing healthcare service provision at the study site. The review team comprised five members from the National TB Program in charge of monitoring and evaluation, two advisors from the regional level, six nurses and data clerks from the Nkembo health facility, and one from the WHO as technical support staff. All of them were briefed on the data reconciliation exercise, the use of templates and the task distribution.

### 2.3. Hypothesis

High TB burden facilities frequently experience challenges related to TB data completeness, accuracy, and consistency. Thus, considering these issues, we formulated the following hypothesis. Information and records contained in patient folders are not fully transcribed into the hospital’s TB registry. Similarly, not all information recorded in the TB registry is transferred into the monthly reports. This situation might contribute to the underreporting of TB cases at the Nkembo health facility.

### 2.4. Sampling

The study employed a progressive and exhaustive sampling approach covering the period from January to August 2023. The sampling unit consisted of recorded patients, who also served as the study units. In this study, all the TB patients registered during the study period in 2022.

### 2.5. Data Collection

A desk review was carried out using TB data recorded in the patient registry of the health facility in comparison with the patient folders.

The variables collected are the number of notified TB cases, the number of TB cases with known HIV status for the 2023 cohort patients and the number of TB patients successfully treated at the end-treatment evaluation for the 2022 cohort. This last indicator was compared to the other issues, which are the number of patients lost to follow-up, the number of deaths, the number of patients whose treatment failed, and the number of patients who were not evaluated.

### 2.6. Data Sources

The key recording and reporting forms for TB are patient folders, laboratory registers, patient registers, laboratory order forms and patient appointment cards. Data from these tools are extracted and used to generate monthly reports, which are aggregated to produce the quarterly report. The patient folder is used to follow up confirmed TB patients, while the laboratory registers capture information on all presumptive cases sent by the clinicians with the laboratory order. All the patients with folders are recorded in the TB register, which is used to prepare the monthly report that is generated at the facility level. At the facility, they have the monthly report for accurate monitoring. After three months, these reports are consolidated in a quarterly report, which is sent to the NTP according to the formal circuit. A validation process is supposed to be done at all the steps.

Prior to creating the dataset, all data retrieved from registers and patients’ folders were fully de-identified by the staff of the Nkembo health facility, who participated in the data reconciliation process but not in data analysis and interpretation.

For the current study, three documents were used as data sources. They are the monthly reports from the Nkembo Health treatment center, the patient registry, and the patients’ folders. The first step was to collect all the patients’ folders in the service archives for the targeted period. And then, the monthly reports and the registry were made available by the TB service at the health facility.

### 2.7. Data Reconciliation

The reconciliation was done in two phases. Firstly, the team started by checking if all the folders were recorded in the register. In the meantime, they checked if all the cells in the registry were filled. If not, they went back into the folders to find out the missing information to fill in the register.

Secondly, the team checked if the register data were the same as in the report. They started with the notified patients, then the HIV result, and then they ended with the treatment result. At the end, all the discrepancies were cleared, and the data were the same between the three documents. At this stage, we had the data before the review, which were in the current report, and the data after reconciliation. The comparison was done between both and the gap was calculated.

### 2.8. Data Analysis

Two templates (Table 1 and Table 2) were prepared for data analysis. The first one for TB notification, including the number of TB cases notified and those for whom their HIV status was known. The second one was for the TB treatment outcome.

In the template, the data from the current report were used to fill the “before the review” column, while the reconciled data were used to fill the “after review” column. The difference between the two columns led to measuring the change and the contribution of the data reconciliation exercise.

Table 1 above helps to compare the notified TB cases and those with known HIV status before and after data review.

Table 2 was used to compare the TB cases successfully treated and those who were unsuccessfully treated before and after the data review. Successfully treated patients include those who were cured and those who have completed the treatment, whereas patients with other treatment outcomes were considered unsuccessfully treated.

For the comparison of TB data before and after the review, we first performed the normality test (Shapiro–Wilk test). Given that the data related to the outcome variables were normally distributed, we performed a paired *t*-test afterward. The analysis was carried out using Stata statistical software version 15 (StataCorp, College Station, TX, USA). The level of significance was set at *p* < 0.05 (double-sided).

### 2.9. Ethical Considerations

The study involved secondary analysis of anonymized routine tuberculosis monitoring and surveillance records from the Nkembo Treatment Center. No identifying information was accessed, no biological samples were collected, and no patients were contacted. All data were reviewed by health personnel already mandated to manage TB data at the facility level. In accordance with the national ethics committee’s guidance—which exempts operational research using fully anonymized programmatic data from formal ethical review—this study did not require Institutional Review Board approval, and informed consent was not applicable.

## 3. Results

### 3.1. Notified Tuberculosis Cases

The variables compared include the number of notified TB cases, the number of notified TB cases with known HIV status and the number of successfully treated TB patients. We compared the outcomes before and after the data reconciliation for each month. The overall results are shown in the table below.

Table 3 shows that the health facility had recorded 2156 TB cases (monthly mean: 269.5 +/− 35.84 cases) from January to August 2023; 730 of them had known HIV status (monthly mean: 91.25 +/− 56.30) and 824 were successfully treated (monthly mean: 103.0 +/− 25.55). After the review, statistically significant increases were observed in the total number of TB cases (2156 vs. 2623; *p* = 0.0003), the number of TB cases with known HIV status (730 vs. 1762; *p* = 0.0017), and the number of TB cases successfully treated (824 vs. 1678; *p* = 0.0001).

**Figure 2:**
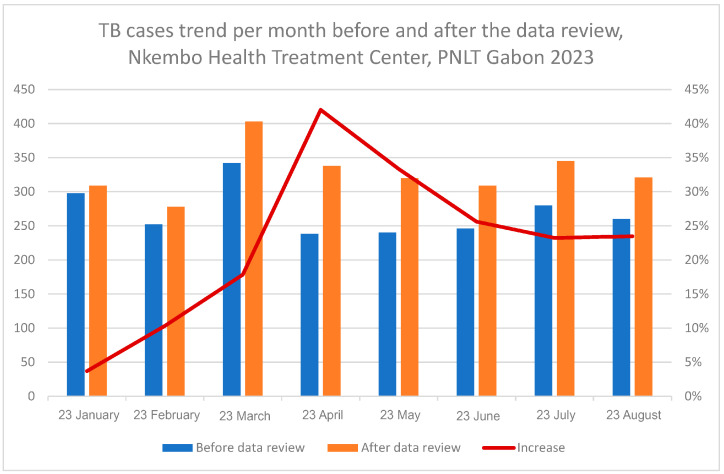
Trend of notified TB cases per month before and after data review, at Nkembo health facility.

Figure 2 shows the trend in the notified TB cases. A substantial increase in the monthly number of TB notifications was observed at the Nkembo health facility after the data review process between January and August 2023. Furthermore, as compared to the situation before the review, April and May of the year 2023 were the months with greater gaps in terms of TB notifications after the data review.

### 3.2. Notified Tuberculosis Cases with Known HIV Status

**Figure 3:**
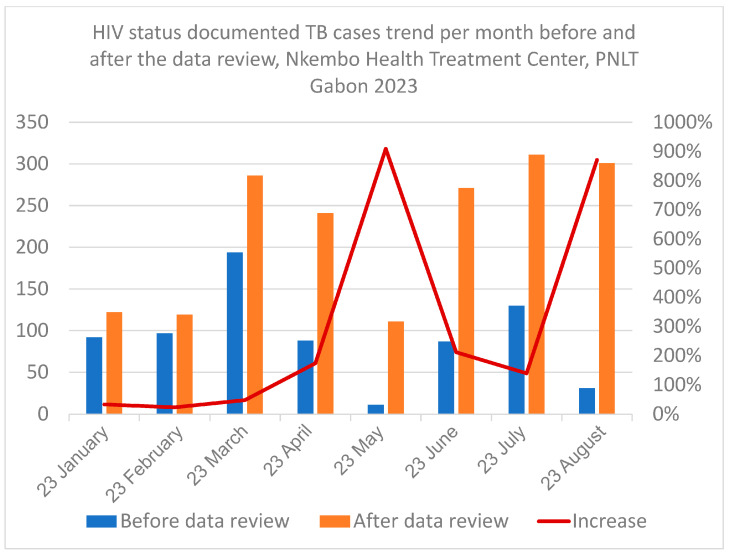
Trend of notified TB cases with known HIV status per month before and after data review at Nkembo health facility.

Figure 3 shows that, compared to the situation before data review, a consistent increase in the monthly number of notifications of TB cases with known HIV status throughout the study period (January–August 2023) was attained after the review process. The gaps were greater between June and August 2023.

### 3.3. Successfully Treated TB Cases

**Figure 4:**
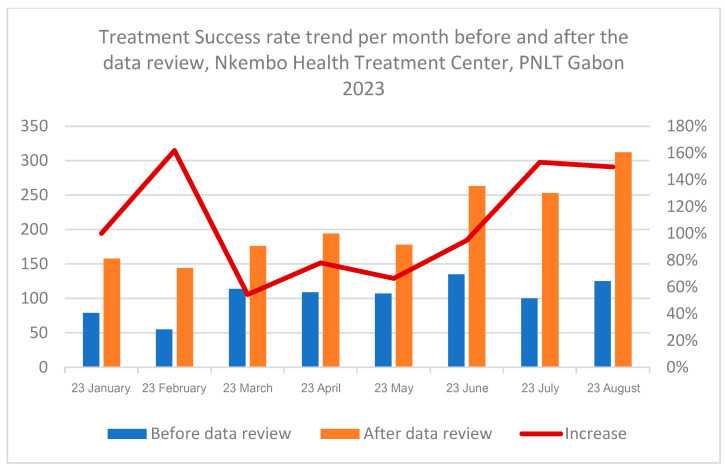
Trend of number of successfully treated cases per month before and after data review at Nkembo health facility.

Figure 4 shows that the number of successfully treated TB cases increased throughout the study period after the data reconciliation process, with substantial monthly increases between June and August 2023.

## 4. Discussion

In this study, a TB data review was conducted at the Nkembo health facility, known as the health setting caring for over 70% of all TB cases in Libreville, Republic of Gabon. The “onion model” was used during the data review to understand the reasons for the underreporting of TB cases in Gabon.

### 4.1. Onion Model

For low-income countries, where the numbers of TB cases reported are considered unreliable due to poor access to health services and/or incomplete case notification, the WHO used three methods until 2015 to estimate TB incidence: (1) the annual risk of infection, (2) the onion model, and (3) prevalence surveys [10].

In this data review, we considered the onion model, which helps not only to estimate the number of cases but also, most importantly, to identify accurate strategies to reach those who are missing. Based on this model, TB cases can be classified into notified cases, cases that were diagnosed but not notified, and undiagnosed cases.

Starting with the number of cases notified by the NTP (the core of the onion), each successive layer of the onion represents the percentage of missed cases, i.e., cases diagnosed within the NTP system but not notified, cases diagnosed outside the NTP system and not notified, cases who accessed health services but for whom a diagnosis was lacking, cases with access to health services who did not use them, and cases with no access to health services. The various layers are determined in order to calculate the proportion of incident TB cases missing from TB notification data and to prioritize the programmatic or health system interventions that might be required to address these missed opportunities for TB notification and/or diagnosis [11].

The Nigerian NTP used the onion model in the “Spot to Tent Onion Model” (STOM) Project in 2023 [12]. Through a grant from the USAID-funded Tuberculosis Implementation Framework Agreement (TIFA) project, implemented by JSI Research and Training Institute, KNCV Nigeria implements the STOM project, which employs a systematic approach of TB contact investigation. The use of the onion model approach helped to increase the contact investigation coverage from 67% to 80%, to boost the community case finding from 8% to 20%, and to scale up TB preventive therapy (TPT) from 2% to 50% of eligible contacts.

### 4.2. Findings from This Study

Our findings confirm the hypothesis that substantial underreporting has undermined the TB program performance at the Nkembo health facility. The program is performing better than the routine reports at the national level. The significant differences observed before and after data reconciliation reflect improvements in data completeness and reporting accuracy. This study demonstrates the need for routine data quality assessments, capacity building and supportive supervision in high-burden health settings. It also highlights potential systemic issues contributing to underreporting. Considering the onion model, notified cases represented 42% in 2022; the review showed that 22% could be caught with the data reconciliation exercise. Thus, we can estimate that the Gabonese TB program can reach approximately 66% of TB cases.

Beyond the TB cases diagnosed but not notified by health settings of the public sector, there are also many other cases from the private sector. This situation is critical given that Gabon is a country where the health system is highly centralized, with many private health facilities closer to the communities. In Nigeria, for instance, the underdiagnosis was compounded by the underreporting of TB cases, primarily from the private sector and particularly in urban health settings, according to the epidemiological review of January 2023 [13].

### 4.3. Data Reconciliation

The data reconciliation exercise in routine surveillance helps to fix data discrepancies between different sources of data. It improves the completeness, accuracy, and timeliness of TB data. It enhances the capacity of health workers to manage and use data. It fosters better program planning, monitoring, and patient care. The three examples below are showing some benefits.

In South Africa, a research team from the Aurum Institute conducted a baseline data reconciliation of drug-resistant TB (DR-TB) records, followed by a data quality improvement process in 2020. After the intervention, data completeness for key variables reached 90–100%, and concordance between clinical folders and the electronic database was 100%. This high-quality data supported regulatory submissions and improved patient management [14]. Furthermore, in South Africa again, when comparing the TB-DR treatment record or patient medical file, the TB Identification Register, and the TB module in TIER.Net at 15 health facilities across the country, Joshua P. Murphy and colleagues observed statistically significant differences in completeness and moderate agreement (Kappa 0.41–0.60) for site of disease, TB treatment outcome, and use of smear microscopy or X-ray as a diagnostic test. They concluded that improvements in the data completeness of TIER.Net compared to the TB Treatment Record are the most urgent area for improvement, especially recording of TB treatment outcomes [15].

The Kenyan TB program has implemented data reconciliation in several counties to assess routine TB service data. The exercise identified gaps in data completeness, accuracy, and timeliness. Findings informed targeted supervision, on-the-job training, and system improvements, resulting in more-reliable data for program planning and monitoring [16].

### 4.4. Strengths and Limitations of the Study

The present work highlights the need to integrate the TB data reconciliation into routine surveillance, especially in the high-burden health facilities, as they may face issues related to data management, especially data completeness, accuracy, and consistency. Compared to the situation before the data review, the reconciliation process resulted in a 22% increase in notified cases. Similarly, the TB cases with documented HIV status rose by 141%, and the successfully treated TB cases increased by 104%.

This study is limited by the fact that some folders were not fully completed, with some missing information. If this exercise is regularly conducted, the health workers can have the possibility to fill in missing data. Additionally, the study did not cover all the onion model layers, which would have been helpful to figure out the underreporting situation in Gabon. Also, the study did not consider including the laboratory step. So only one layer, the third one, was covered by the study. It is related to the cases diagnosed but not reported. The three indicators analyzed are part of this layer. Finally, gathering some qualitative data could be important to analyze the reasons for this data mismanagement.

## 5. Conclusions

The study findings suggest that data reconciliation effectively enhances TB surveillance, and data completeness in particular, in high-burden areas, thus improving TB program performance. Future research should delve deeper into the public health implications of these findings.

## Figures and Tables

**Table 1:** TB case notification for the period before and after the data review.

**Before the Review**			**After Reconciliation**	**After the Review**	
	**Monthly (2023)**	**%**		**Monthly (2023)**	**%**
Number of notified TB cases (2023 cohort)					
Number of TB cases with known HIV status					

**Table 2:** Treatment outcome for the period before and after the data reconciliation (2022 cohort).

**Before the Review**			**After Reconciliation**	**After the Review**	
**Indicator**	**Monthly (2022)**	**%**		**Monthly (2022)**	**%**
Number of notified TB cases (cohort 2022)					
Number of cured patients					
Number of patients who completed the treatment					
Number of patients lost to follow-up					
Number of TB patients who died					
Number of TB patients with treatment failure					
Number of patients not evaluated					
Number of TB patients successfully treated					

**Table 3:** Number of TB cases, TB patients with known HIV status and patients successfully treated before and after data review.

	Nb TB Cases	With Known HIV Status	Successfully Treated
Before data review	2156	730	824
After data review	2623	1762	1678
Increase	22%	141%	104%
*p*-value	0.0003	0.0017	0.0001

Notes: Nb, number; HIV, human immunodeficiency virus; TB, tuberculosis.

## Data Availability

Data are available in the National Tuberculosis Program, M&E office.
